# Sustainability and Versatility of the ABCDE Protocol for Stress Echocardiography

**DOI:** 10.3390/jcm9103184

**Published:** 2020-09-30

**Authors:** Eugenio Picano, Angela Zagatina, Karina Wierzbowska-Drabik, Clarissa Borguezan Daros, Antonello D’Andrea, Quirino Ciampi

**Affiliations:** 1 Biomedicine Department, CNR Institute of Clinical Physiology, 56124 Pisa, Italy; 2 Cardiology Department, Saint Petersburg State University Clinic, Saint Petersburg State University, 199034 Saint Petersburg, Russia; zag_angel@yahoo.com; 3 First Department and Chair of Cardiology, Bieganski Hospital, Medical University, 90926 Lodz, Poland; wierzbowska@ptkardio.pl; 4 Cardiology Division, Hospital Sao José, Criciuma 88800000, Brazil; clarissabdaros@cardiol.br; 5 Cardiology Division, General Hospital, 84014 Nocera Inferiore, Italy; antonellodandrea@libero.it; 6 Cardiolody Division, Fatebenefratelli Hospital, 82100 Benevento, Italy; qciampi@gmail.com

**Keywords:** coronary artery disease, functional test, heart failure, stress echo, sustainability

## Abstract

For the past 40 years, the methodology for stress echocardiography (SE) has remained basically unchanged. It is based on two-dimensional, black and white imaging, and is used to detect regional wall motion abnormalities (RWMA) in patients with known or suspected coronary artery disease (CAD). In the last five years much has changed and RWMA is not enough on its own to stratify patient risk and dictate therapy. Patients arriving at SE labs often have comorbidities and are undergoing full anti-ischemic therapy. The SE positivity rate based on RWMA fell from 70% in the eighties to 10% in the last decade. The understanding of CAD pathophysiology has shifted from a regional hydraulic disease to a systemic biologic disease. The conventional view of CAD encouraged the use of coronary anatomic imaging for diagnosis and the oculo-stenotic reflex for the deployment of therapy. This has led to a clinical oversimplification that ignores the lessons of pathophysiology and epidemiology, and in fact, CAD is not synonymous with ischemic heart disease. Patients with CAD may also have other vulnerabilities such as coronary plaque (step A of ABCDE-SE), alveolar-capillary membrane and pulmonary congestion (step B), preload and contractile reserve (step C), coronary microcirculation (step D) and cardiac autonomic balance (step E). The SE methodology based on two-dimensional echocardiography is now integrated with lung ultrasound (step B for B-lines), volumetric echocardiography (step C), color- and pulsed-wave Doppler (step D) and non-imaging electrocardiogram-based heart rate assessment (step E). In addition, qualitative assessment based on the naked eye has now become more quantitative, has been improved by contrast and based on cardiac strain and artificial intelligence. ABCDE-SE is now ready for large scale multicenter testing in the SE2030 study.

## 1. Conventional Stress Echocardiography: Strengths and Pitfalls

**Figure 1:**
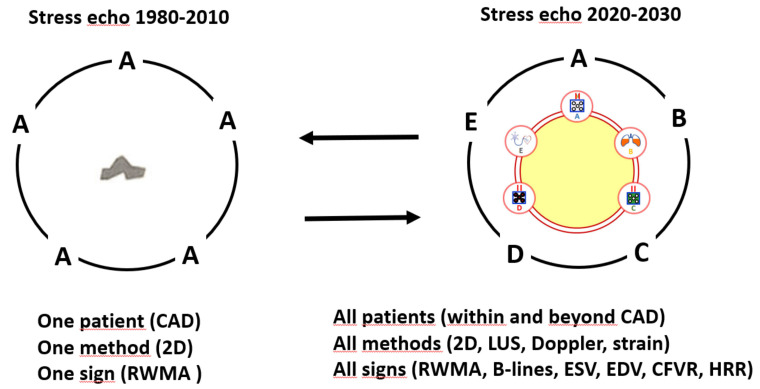
The conceptual approach of advanced stress echocardiography (SE): from vulnerable stenosis to vulnerable patient. Left panel: in the classical or conventional approach that has been used for 40 years and in first-generation multicenter studies such as Echo-Persantine Cooperative (EPIC) and Echo-Dobutamine Cooperative (EDIC) studies, SE was only focused on the hemodynamic significance of the coronary stenosis. Right panel: the advanced ABCDE protocol used in the last 5 years in second-generation SE2020 and SE2030 multicenter studies. The focus is shifted to the patient as a whole and involves the assessment of vulnerability to ischemia (step A with RWMA is now corroborated by regional and global longitudinal strain and artificial intelligence), pulmonary congestion (step B), the left ventricular contractile and preload reserve (step C), coronary microcirculation (step D) and chronotropic reserve (step E). CAD—coronary artery disease; 2D—2-dimensional echocardiography; RWMA—regional wall motion abnormalities; LUS—lung ultrasound; ESV—end-systolic volume; EDV—end-diastolic volume; CFVR—coronary flow velocity reserve; HRR—heart rate reserve.

Stress echocardiography (SE) is based on the assessment of inducible regional wall motion abnormalities (RWMA) for the functional evaluation of patients with known or suspected coronary artery disease (CAD). It has had a well-established clinical role for several decades [1] and is now well recognized in guidelines and recommendations as a first-line imaging test [2,3]. In the last 20 years, the utilization rate of SE has increased by 5- to 10-fold in risk- and cost-sensitive environments due to the increase in the awareness of patients, doctors and payers of the importance of economic costs and long-term radiation risks [4,5,6]. The unique versatility of SE also allows it to be applied outside of CAD in conditions such as valvular heart disease, congenital heart disease, dilated or hypertrophic cardiomyopathy, extreme physiology and post-heart transplant rejection [7,8]. SE is considered to be a well-established technique but this strength is also its major limitation because it has remained conceptually and methodologically unchanged since it was introduces 40 years ago (see Figure 1, left panel). The methodology uses two-dimensional echocardiography technology for patients with known or suspected CAD and involves the assessment of RWMA with the naked eye. This approach has obvious merits since it provides a highly specific marker of myocardial ischemia and underlying epicardial CAD, which provides the ability for excellent risk stratification, it has an outstanding safety record even with pharmacological stress and contrast, and an unsurpassed cost-benefit profile [9,10]. However, there are several limitations. The reliability of the test is dependent upon the acoustic window [11]. The interpretation of RWMA is subjective and qualitative [12,13], and the level of experience required by guidelines for professional accreditation and certification of competence [14,15] does not guarantee reading accuracy and harmonization of the reading criteria [16]. The SE positivity rate based on RWMA fell from 70% in the eighties to 10% in the last decade for all tests based on myocardial ischemia, most likely due to increased use of anti-ischemic therapy at the time of testing [17]. Its diagnostic sensitivity and prognostic negative predictive value are suboptimal, for example, its predictive value is lower than that of a test based on perfusion imaging [18,19] and has declined over the last 40 years [20]. All of the limitations and weaknesses of the SE technique can now be fixed or minimized with the use of state-of-art technology and protocols. Contrast enhances the image quality, improves the reproducibility of RWMA assessment and reduces the number of unreadable studies to <1% [21,22]. Cardiac strain can be measured quantitatively by assessing global longitudinal strain. The assessment of regional strain can also be useful in apical and middle segments, since not all segments were created equal. This can be done not only by visual estimation but also with deformation imaging [23,24]. Most importantly, the state-of-the-art ABCDE-SE is able to evaluate not only coronary plaque but it can also comprehensively assess patient vulnerabilities by taking advantage of the extraordinary versatility of the technique. The old landline telephone has now been replaced by a mobile smart-phone with a variety of applications that make it well-suited to tailor the right test at the right time for the right patient [25].

Some aspects of the ABCDE protocol have already been discussed in previous review papers [26]. However, the field is evolving so rapidly that both the methodology and the understanding of the method is being reshaped on the basis of new evidence and new experiences. For example, the so-called quadruple imaging (ABCD) protocol was changed to incorporate the non-imaging component E as an integral part of the core protocol. This review will present the updated view of the protocol while paying special attention to evidence gathered in the last two years and the perspectives of new studies that are underway.

## 2. ABCDE-SE: Rationale, Methodology, Results

In the novel state-of-the-art ABCDE SE protocol, RWMA remains the first and main step A, which is corroborated by quantitative deformation imaging of global and regional strain. B-lines are assessed in step B, the left ventricular contractile and preload reserve in step C, Doppler-based coronary flow velocity reserve (CFVR) in the left anterior descending coronary artery or contrast myocardial perfusion imaging is assessed in step D, and ECG-based assessment of heart rate reserve in non-imaging takes place in step E. The five parameters converge conceptually, logistically and methodologically in the ABCDE protocol, which can be applied to all stresses and all patients and it allows a comprehensive color-coded risk stratification of the vulnerable patient beyond coronary artery stenosis (Figure 1, right panel). A normal ABCDE-SE response identifies a “non-ischemic”, “dry”, “strong”, “warm” and “fast” heart at very low risk. An abnormal ABCDE-SE response identifies an “ischemic”, “wet”, “weak”, “cold” and “slow” heart, with RWMA, pulmonary congestion, dilated end-systolic volume, reduced CFVR and blunted chronotropic response [26]. 

The assessment of lung water by lung ultrasound and B-lines is an extension of resting transthoracic echocardiography, which was introduced in cardiology in 2004 [27] and since then it has been extensively applied for diagnostic and prognostic purposes, mainly in heart failure patients [28,29,30]. The B-step was proposed for scanning at rest and during stress [31] with the 28-site scan requiring several minutes of imaging and analysis time; however it is more suitable for research than for clinical applications, especially during stress when time is short and there are many things to observe. The ideal scanning protocol is the simplified 4-site scan, which is as accurate as the 28-site scan but much easier to obtain and can be analyzed in <1 min. This usually takes place once the test is finished since lung water takes a few minutes to disappear after the end of exercise or antidote administration in the case of pharmacological stress [32]. Each site is scored from 0 (horizontal A-lines in black lung) to 10 (coalescent B-lines in white lung). The scores of the individual sites are added up and the final cumulative score may range from 0 or 1 (normal) to 40 (alveolar pulmonary edema). The peak stress scores are quantified as normal (0 to 1), mildly abnormal (2–4), moderate (5–9) or severe (≥10) [33]. The number of B-lines is related to the amount of extravascular lung water, which ranges from normal values (<500 mL) to mild (500–1000 mL), moderate (1000–1500 mL), and severe (>2000 mL) depending on the degree of accumulation [34]. The number of stress B-lines are a better reflection of the cardiac natriuretic peptide concentration and peak anaerobic threshold that rest B-lines. This is not surprising since interstitial lung water accumulation during exercise compromises the gas exchange capability [35]. Stress B-lines appear in one out of three patients without B-lines at rest [35]. More peak stress B-lines indicate a worse prognosis [35,36,37], and they can be found in all situations associated with dyspnea of cardiac origin [38], from CAD to diastolic dysfunction, from heart failure with reduced ejection fraction to heart failure with preserved ejection fraction [39,40], from aortic insufficiency to mitral stenosis [41] or extreme physiological settings such as trekking in high altitude [42] or apnea diving [43]. If there is water, there is a defect somewhere in the cardiovascular chain linking the alveolar capillary barrier to cardiovascular performance [44]. The cutoff value for abnormal B-lines is a rest or stress value of ≥2 units and this is the same for all stresses. A normal heart without B-lines is called dry, while an abnormal heart with B-lines is referred to as wet.

Volumetric echocardiography is the tool used for assessment in step C [45]. The biplane Simpson’s rule method is used to measure the left ventricular volumes from apical four-chamber and two-chamber views. When the two orthogonal views are not obtainable, an apical single chamber view is sufficient [46]. When neither apical views are obtainable in non-distorted ventricles, a parasternal view with volume measurements by the Teichholz rule is enough to obtain a reliable estimate of left ventricular volume variations from rest to stress [47]. The feasibility of the method increases with contrast injection, which enables better endocardial contour recognition [48]. The method can be applied with shorter imaging time and greater accuracy without depending on the geometric assumptions of real-time three-dimensional echocardiography, which is currently limited by low frame rates and large transducer footprints. The image acquisition is the same as for RWMA, and the analysis time is the same as that required for the ejection fraction, which is provided as part of the minimum data set of SE. However, end-systolic volume and end-diastolic volume are analyzed as a part of two different and equally important aspects of left ventricular function and form [49]. In fact, end-systolic volume is an index of function, and end-diastolic volume is an index of form [50]. The physiological companion of end-systolic volume is systolic blood pressure measured by cuff sphygmomanometer to obtain force or elastance. Left ventricular contractile reserve (LVCR) is the stress to rest ratio and is an index of contractility; it is conceptually different from the ejection fraction since it is independent of preload, heart rate and afterload changes. Rather, it is a pure index of LVCR whereas the ejection fraction is a hybrid index combining a functional index such as end-systolic volume with a morphologic index such as end-diastolic volume. Therefore, it is simpler to obtain than the ejection fraction since only the end-systolic volume (more reproducible than end-diastolic volume) is required in addition to systolic blood pressure, which is already a part of the minimum data set provided by SE. Therefore, the end-systolic volume provides an index of systolic function and contractile reserve and can be obtained during exercise, noninvasive pacing, dobutamine or dipyridamole SE. For any given systolic blood pressure, the smaller the end-systolic volume, the better the LV contractile reserve and outcome [51]. For any given ejection fraction value at rest and during stress, the prognosis is clearly worse with lower values of LVCR. The cutoff values for abnormal LVCR are lower (<1.1) for vasodilators [52,53] and higher (<2.0) for stressors such as exercise and dobutamine, which have a stronger inotropic effect. A normal heart with preserved LVCR based on force is referred to as strong, while an abnormal heart with reduced LVCR is known as weak.

At low heart rates, the end-diastolic volume normally increases in response to stress, but beyond 100–110 bpm the diastolic time is reduced and further filling augmentation is precluded [54]. Therefore, during exercise or dobutamine use, the end-diastolic volume response should be assessed at the intermediate stages of exercise while the response to vasodilators can be evaluated at peak stress. The normal response is an increase in end-diastolic volume of around 10% at the intermediate stages of exercise [54]. The normal preload reserve is equally as important as the contractile and the chronotropic reserve to guarantee an effective increase in cardiac output. A normal heart with preserved preload reserve based on the increase in end-diastolic volume is called compliant, an abnormal heart with reduced preload reserve is called stiff.

Step D is based on the color- and pulsed-wave Doppler evaluation of the coronary flow velocity reserve (CFVR) in the mid-distal left anterior descending coronary artery [55]. CFVR is simply calculated as the peak/rest values of the peak diastolic coronary flow velocity [56]. A readable signal is obtained in >90% of consecutive cases, and contrast is needed in <5% of cases to enhance the signal. The feasibility is >95% with vasodilators, but it is also excellent with pacing and dobutamine, and it is >80% even with exercise if a semi-supine bicycle is used and flow monitoring is obtained in the early steps of exercise [57]. A reduced CFVR in the absence of RWMA is very frequent in the presence of angiographically normal coronary arteries and indicates abnormal coronary microcirculatory function, which predicts the recurrence of ischemia, development of heart failure and cardiac and non-cardiac death [58]. A reduced CFVR is associated with low-grade inflammation, which is probably a universal mechanism of disease that is also found in some forms of cancer, dementia and other chronic inflammatory conditions [59]. The heart serves as physiological paradigm, since it increases in thickness, stiffness, function and temperature with increased flow [60]. A normal heart with preserved CFVR is called warm, an abnormal heart with reduced CFVR is called cold.

Step E is based on ECG, which evaluates the heart rate reserve as the peak/rest value of the heart rate derived from a 12-lead ECG or simply from the ECG lead that is automatically read in the echo monitor. It is an index of cardiac sympathetic reserve, is obtainable in all patients with 100% success rate and reproducibility. It is an index of cardiac autonomic function, and in particular, of cardiac sympathetic reserve, which can be impaired by several conditions other that CAD such as heart failure or hypertrophic cardiomyopathy [61]. It is also a biomarker of cardiac autonomic dysfunction and as such, mainly predicts sudden cardiac death and cardiac arrhythmias [62]. Chronotropic stress is higher with exercise and dobutamine and milder with vasodilator stress, which also elicits an adrenergic response unrelated to a decrease in blood pressure or inducible ischemia [63,64,65]. The cut-off for abnormal values are higher with exercise and dobutamine (<1.80) than with vasodilators (<1.22) and are maintained in populations using beta-blockers, in whom baseline heart rate is lower but the frequency response is preserved. The prognostic value of a reduced heart rate reserve has been shown to be independent of inducible RWMA with exercise [66], dobutamine [67] and dipyridamole [68]. With dipyridamole stress, the abnormality cut-off associated with a worse outcome is slightly lower (1.17) in the presence of chronic atrial fibrillation [69] and provides incremental prognostic information over RWMA and CFVR [70]. 

**Figure 2:**
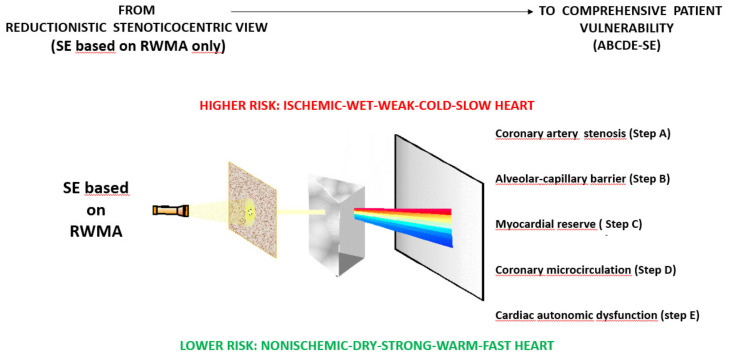
The prognostic approach with advanced SE. Left panel: in the classical or conventional SE approach risk stratification is based only on the presence and extent or regional wall motion abnormalities, which offers a limited measure of risk. Right panel: the advanced ABCDE protocol acts as a Newton’s prism that offers a multi-dimensional response that includes regional wall motion, pulmonary congestion, preload and afterload reserve, coronary microcirculation, and sympathetic reserve. Each color identifies a specific vulnerability and is a possible target for selective therapeutic interventions.

Much has changed as a result of this methodological and conceptual remodeling. The pathophysiological model has shifted from stenosis vulnerability to patient vulnerability. The prediction potential has shifted from the recurrence of angina to all events including cardiac death, non-cardiac death, arrhythmic events and progression to heart failure. Risk stratification has become multi-dimensional and color-coded (Figure 2), and thus, it more realistically addresses the need for tailored functional characterization, risk assessment and SE-driven therapy in the age of personalized medicine.

## 3. The Limitations of the ABCDE Protocol

Not all the problems of cardiology are solved with the ABCDE protocol. Yet, it is a simple way to capture a more comprehensive view than was possible in previous decades of the clinical use of SE. Its application is more suitable for risk stratification and functional characterization than for primary diagnosis of CAD. In general, the presence of multiple positive biomarkers points to more extensive anatomic CAD in patients with chronic coronary artery disease, but in principle, each of the four new markers can be present due to a variety of causes that are independent of the presence and severity of underlying CAD.

Not all questions can be answered with the use of this protocol. However, the improvement in clinically relevant information in the last five years did not come from a technological upgrade or industrial innovation, but rather from a more integrated pathophysiological approach to the complexity of the cardiac patient. Strain is useful, contrast helps, and three-dimensional imaging is the unavoidable future, however, the conceptual and methodological framework of ABCDE can help to exploit these technological innovations in a more clinically-oriented way.

More data and information are required for clinical use. In particular, we need to know whether each letter or level of the protocol predicts specific, individual endpoints, for instance, acute decompensated heart failure with step B or cardiac arrhythmias and sudden death with step E. To achieve this critical mass of data, large scale effectiveness studies are needed and are currently under way with the SE 2020, and the SE 2030 study which is about to start.

Last but not least, the application of the new ABCDE protocol should always start from a clinical question or suspicion. The pathophysiological processes that underlie the protocol are always at work, but not all of them are always relevant for the treatment or interpretation of symptoms in the individual patient. Whether the steps in the protocol are essential or not may well depend on the different type of patient and the scope of testing that is required, including the primary diagnosis of CAD, identification of functional mechanisms of disease and symptoms, risk stratification, guide to therapy, or objective assessment of therapy efficacy.

Apart from the limitations of the protocol as a whole, there are specific pitfalls in each step that should be considered, and which can possibly be solved by technological and cultural advances in the near future.

## 4. Pitfalls of Individual ABCDE Steps

**Step A.** SE has several obvious disadvantages. The three main disadvantages are patient-related (that is, it depends on the acoustic window), operator-related (depends on the reader’s expertise), and physiology-related (the complexity of the patient’s physiologically cannot be reduced to a critical epicardial coronary stenosis and inducible RWMA). The inability to establish a good acoustic window precludes testing in a limited proportion of patients, but with the use of contrast and last-generation technology this percentage shrinks to <1%, even with the current morbid obesity pandemic. The subjective reading of RWMA by the naked eye requires training and does not allow improvisation. The training requirement for professionals in this field has been established as at least 100 studies per year with an expert supervisor to gain certification, and 100 studies per year to maintain accreditation.

**Step B.** Lung rockets reflect the presence of an interstitial syndrome, which can be due to water, inflammation or fibrosis. B-lines must not be mixed up with Z-lines, which are frequently observed as bundle-shaped reflections arising from the pleural line, but unlike true B-lines, they do not erase A-lines, are ill-defined, less echogenic than the pleural line, are short and do not move in synchrony with respiration. The training requirement for professionals are easier than for RWMA and have been established as <50 studies per year with an expert supervisor to gain certification.

**Step C.** The image quality required for consistent quantitative measurements of LV volumes is higher than that needed for RWMA. The delineation of LV volumes can be time-consuming on unenhanced images, and calculations are faster and more repeatable with contrast-enhancement or artificial intelligence-based software, which is now on board commercially available machines. The professional training requirements are the same as for RWMA, but the measurements of LV volumes may lack repeatability when images are of sub-optimal quality.

**Step D.** The measurement of CFVR in LAD as a proxy for global coronary flow reserve is attractive for its simplicity but it has conceptual and practical limitations. The variations in flow velocity are proportional to the total blood flow if the diameter of the vessel lumen is kept constant. In reality, the diameter of epicardial coronary arteries increases by an average of 30% in healthy subjects following adenosine infusion Therefore, failure to take into account epicardial coronary artery vasodilation during hyperemia may cause a nonsystematic underestimation of coronary flow reserve, which can be more accurately calculated by the velocity-time integral and the cross-sectional area. CFVR assessment in the right coronary artery and left circumflex is also feasible but imaging time is too long and the technical difficulties are greater, and therefore, the clinical assessment of CFVR is restricted to one single coronary district even though CAD is a regional and multi-district disease. The feasibility of LAD flow imaging is extremely high, although 5% of patients remain unreadable even after contrast enhancement. The professional training requirements are the same as for RWMA, but the interpretation is much more quantitative and easier. Coronary flow is more difficult to obtain but easier to interpret than RWMA.

**Step E.** If atropine is added to dobutamine or dipyridamole, the cutoff values may be higher and are not validated. During pharmacological testing, the value of heart rate reserve is not affected by physical conditioning and deconditioning, which affects the value of chronotropic incompetence during exercise. The peak heart rate cannot be recorded at a fixed time corresponding to the end of adenosine or dipyridamole infusion, since the greatest change in heart rate (measured each minute) occurs in the time interval from 0 to 5 min after drug infusion. It is important to record the peak value since there is minimal interpatient variability, whereas the peak value always corresponds with peak stress during exercise. Beta-blockers reduce the absolute rest and peak values of heart rate, but the relative rest-stress changes mirrored in heart rate reserve are essentially the same. No specific training is required because the heart rate is given in real time and automatically by any machine.

## 5. Comparison with Other Imaging Techniques in Different Clinical CAD Scenarios

As with any new technique, there is a lack of direct comparative data with other imaging techniques in different clinical CAD scenarios. If the ABCDE replaces the conventional SE protocol as the new standard, this gap could be filled quickly. In line with this principle, some of the ABCDE steps can be incorporated in other non-ultrasound cardiac functional stress testing techniques. Step E might include perfusion data from radionuclide myocardial perfusion imaging, which can also estimate LV volumes in step C, although with less temporal and spatial resolution. Cardiac magnetic resonance is ideally suited to obtain information for step C with LV volumetric imaging and for step D with myocardial perfusion imaging. Cardiac functional testing is an alternative approach to anatomic imaging with noninvasive cardiac computed tomography angiography, that is recommended in current guidelines for patients with low pre-test probability of disease. However, this approach may change when sustainability considerations are incorporated into decision-making, as a more comprehensive, less stenosis-centered approach gains popularity in the field of cardiac imaging and in line with the pathophysiological lessons learned from COURAGE, ORBITA and ISCHEMIA trials.

## 6. The Sustainability of SE

The growth of SE in recent years is a result of the dramatically changed cultural milieu surrounding health care delivery. The concept of sustainability has emerged as a key feature in shaping the all health care practices including cardiac imaging [71]. Sustainability is also a concept that includes at least four components and involves at least four stakeholders. The first component is diagnostic accuracy, which measures the medical value of the test and which are important because missed diagnoses imply extra-risk and the extra-cost of further downstream examinations. The accuracy of SE is high and is comparable to other available techniques.

The second component is the direct cost. SE is considerably less expensive than other alternative imaging techniques, and economically, a test with similar accuracy and higher cost would not be considered a viable option, although the situation is different in health care in that those who buy usually do not pay. With the changing economic climate, there is less societal and political tolerance for the delivery of high cost and low value health care.

The third component is safety. SE has an excellent safety profile since stress can be interrupted as soon as RWMA appear during real-time monitoring, contrast has excellent tolerability and non-ionizing radiation is used. Prioritizing safety is also important to minimize the legal concerns connected with iodine contrast nephrotoxicity. Zero radiation is essential to mitigate the cancer burden due to cumulative radiation exposure, and in Europe the law reinforces the use of radiation-free options when available [72].

The fourth component is the environmental footprint due to carbon dioxide emissions. Echocardiography produces 2 or 3 kg of carbon dioxide emissions per examination, which is 100-fold lower than magnetic resonance cardiac imaging and is by far the lowest of all the alternative techniques [73]. The cardiac imaging footprint affects climate change, and air pollution and climate change are a strong risk factor for acute and chronic cardiac events [74].

The concept of sustainability is making its way into health care, and cardiac imaging is progressively affected by sustainability issues. Small individual costs, risks, and ecological footprints multiplied by billions of examinations worldwide become a significant societal cost, population risk and environmental burden. There are four stakeholders in the imaging field: the patient, the physician, the payer and the planet [75]. SE is good for all four of these stakeholders, and it will be increasingly difficult to prescribe and practice examinations while ignoring the principles of achieving high value care, zero radiation, low risk, and climate-neutral options whenever possible.

## 7. Perspectives: SE2030

The life of SE began 40 years ago and now life begins again at forty. We need effectiveness studies that are based on real patients, real instruments and real doctors to obtain true and realistic results on the performance of new tests or technologies [76,77,78,79].

ABCDE-SE was the result of the SE2020 study [80] and is the basis of the SE2030 study that is about to get underway. SE2030 is an international, multicenter, effectiveness study and will focus on a spectrum of conditions within and beyond CAD, from all-comers to suspected or known CAD to post-COVID-19 cardiological surveillance, from heart failure with preserved ejection fraction to hypertrophic cardiomyopathy, from valvular heart disease to status post-radiotherapy, from suspected coronary vasospasm to extreme physiology with outdoor SEs performed with pocket size instruments. One project will be devoted to cardiac strain and artificial intelligence to establish the transition from qualitative naked eye to quantitative automated assessment of regional wall motion, in order to solve the currently limitation of strain inter-vendor variability and to consider segmental heterogeneity during stress [81,82]. In particular, artificial intelligence potentially provides a solution for automated and in-depth handling of imaging information, by making measurement objective where it is currently made by the naked eye (such as RWMA in step A) or by hand measurement (such as the calculation of LV volumes in step C).

The new core ABCDE protocol [82] will be supplemented in selected patients by additional parameters, for example, by including F for regurgitant flows [83], G for transvalvular and intraventricular gradients [84], L for left atrial volume and function [85], P for pulmonary and LV end-diastolic pressures [86,87], and R for right ventricular function [88]. A whole new “alphabet” will allow SE to take advantage of its extraordinary versatility, and pave the way for tailored treatment, since each parameter unmasks an important biomarker, a pivot of disease, a potential therapeutic target, and a specific vulnerability in the patient that contributes to prognosis. The test is fully in the cardiologist’s domain, it allows a holistic and tailored view of the patient, exercise can be easily replaced by pharmacological stress to minimize the personal protection needed in times of high epidemiological viral pressure [89] and its safety profile, simplicity, climate-friendly and radiation-free testing make it most appropriate in the quest for sustainability. Its safety and versatility also allow SE to be used to assess the effects of therapy, from anti-ischemic drugs to mechanical coronary revascularization, in reducing the presence and severity of stress-induced RWMA [90].
